# Differences and Similarities of Photocatalysis and Electrocatalysis in Two-Dimensional Nanomaterials: Strategies, Traps, Applications and Challenges

**DOI:** 10.1007/s40820-021-00681-9

**Published:** 2021-07-15

**Authors:** Weiqi Qian, Suwen Xu, Xiaoming Zhang, Chuanbo Li, Weiyou Yang, Chris R. Bowen, Ya Yang

**Affiliations:** 1 Beijing Key Laboratory of Micro-Nano Energy and Sensor, CAS Center for Excellence in Nanoscience, Beijing Institute of Nanoenergy and Nanosystems, Chinese Academy of Sciences, Beijing, 101400 People’s Republic of China; 2 Optoelectronics Research Center, School of Science, College of Life and Environmental Sciences, Minzu University of China, Beijing, 100081 People’s Republic of China; 3 Institute of Materials, Ningbo University of Technology, Ningbo, 315016 People’s Republic of China; 4 Department of Mechanical Engineering, University of Bath, Bath, BA2 7AK UK; 5 School of Nanoscience and Technology, University of Chinese Academy of Sciences, Beijing, 100049 People’s Republic of China; 6 Center on Nanoenergy Research, School of Physical Science and Technology, Guangxi University, Nanning, 530004 People’s Republic of China

**Keywords:** 2D nanomaterials, Photocatalysis, Electrocatalysis, Electrochemistry, Photoelectrochemistry

## Abstract

Photocatalysis and electrocatalysis have been essential parts of electrochemical processes for over half a century. Recent progress in the controllable synthesis of 2D nanomaterials has exhibited enhanced catalytic performance compared to bulk materials. This has led to significant interest in the exploitation of 2D nanomaterials for catalysis. There have been a variety of excellent reviews on 2D nanomaterials for catalysis, but related issues of differences and similarities between photocatalysis and electrocatalysis in 2D nanomaterials are still vacant. Here, we provide a comprehensive overview on the differences and similarities of photocatalysis and electrocatalysis in the latest 2D nanomaterials. Strategies and traps for performance enhancement of 2D nanocatalysts are highlighted, which point out the differences and similarities of series issues for photocatalysis and electrocatalysis. In addition, 2D nanocatalysts and their catalytic applications are discussed. Finally, opportunities, challenges and development directions for 2D nanocatalysts are described. The intention of this review is to inspire and direct interest in this research realm for the creation of future 2D nanomaterials for photocatalysis and electrocatalysis.

## Highlights

- This review focuses on the differences and similarities of photocatalysis and electrocatalysis in the latest 2D nanomaterials.
- Strategies and traps for performance enhancement of 2D nanocatalysts are highlighted.
- Challenges, future directions and applications for new photocatalysis and electrocatalysis exploiting 2D nanomaterials are suggested.

## Graphical abstract

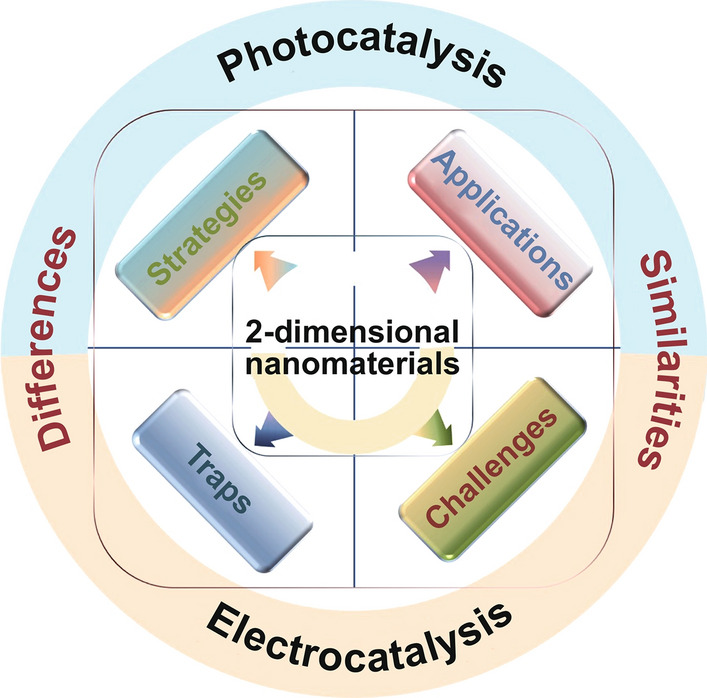

## Introduction

Developing new forms of renewable energy generation can be considered the most promising strategy to tackle the world’s growing environmental challenges and the global energy crisis [1–5]. Catalysis has received intensive interest in sustainable energy development and environmental remediation since the work of Fujishima et al. in 1972, due to their pioneering work on titanium dioxide (TiO_2_) photoelectrodes [6]. Generally, *photocatalysis*, as one of common catalysis, is the utilization of semiconductor photocatalysts to accelerate photochemical reactions, where the photogenerated separated electron–hole pairs participate in the following oxidation–reduction reactions [7–9]. *Electrocatalysis* is a specific form of catalysis that accelerates charge transfer between the electrodes and the electrolyte interfaces [10, 11], where most commonly electrocatalysts are a kind of catalysts attached on the surface of electrodes or as the electrode surface that are largely beneficial for electron transfer between reactants and electrodes [12–14]. So far, photocatalysis and electrocatalysis are often essential parts of chemical processes for water splitting and pollution treatment, which are important reactions for harvesting ubiquitous forms of ambient energy [15–23].

As the gradually deepening process of *two-dimensional* (2D) nanomaterials in molecular design and synthesis [24–29], a number of 2D nanomaterials have been used as a catalyst in their three-dimensional (3D) bulk form; however, their performance as a photocatalysis or electrocatalysis continues to suffer from a low efficiency in terms of charge separation and low transfer kinetics compared to 2D nanomaterials [12, 30–32]. As an example, the traditional design of graphitic carbon nitride (g-C_3_N_4_)-based materials has considered bulk nanosheets [33]. However, due to the strong stacking forces between atom layers, the use of a bulk nanomaterial leads to a low surface reactivity, a high probability of charge recombination and poor solar absorptivity [34, 35]. It can therefore be assumed that the dimensionality and surface characteristics play an important role in determining the key catalytic properties for practical applications and the optimum fabrication process of the material [12]. Hence, research that aims to develop atomically thin 2D catalysts with enhanced charge carrier dynamics and improved mobility continues to attract interest.

To enhance the photocatalytic and electrocatalytic performance, growing attention has been attached to the development of 2D nanomaterials with good electrical conductivity and large surface area [34]. In contrast to conventional 3D bulk nanomaterials, these atomically thin 2D nanomaterials have attracted attention in environmental and energy-related research sectors as a result of their extraordinary stability and activity, often on account of their high specific surface area, robust mechanical structure and excellent electrical conductivity. In addition, 2D nanomaterials have been pursued as economical alternatives to more expensive precious metals, such as platinum and rhodium [35]. Recent progress in multiple atomically thin 2D nanomaterials has broken new ground; there have been rapid developments in the synthesis of 2D nanomaterials, and their resulting properties, surface chemistry and catalytic applications [36]. To date, a detailed understanding on the rational design and construction of efficient 2D nanomaterial-based catalysts as well as the issues associated with industrial scale applications is still not comprehensive enough [37]. Therefore, comprehensive overview is still needed to provide new insights on the fabrication and application of recent developments, and fundamental studies are needed for clear reaction processes to improve catalytic performance for applications that are ripe for industrial exploitation [38–40]. There have been a variety of excellent reviews on 2D nanomaterials for catalysis [10, 34, 41–44]. However, related issues of differences and similarities between photocatalysis and electrocatalysis in 2D nanomaterials are still vacant, but worthy of great attention since demands to generate exceptional catalytic activities are strongly different for photocatalytic and electrocatalytic reaction systems.

Herein, a comprehensive overview will focus on the differences and similarities of photocatalysis and electrocatalysis in the latest 2D nanomaterials. A comparison of differences and similarities of photocatalysis and electrocatalysis in 2D nanomaterials is concluded in Table 1. We will begin with strategies for performance enhancement of 2D nanocatalysts as a highlight, which will point out the differences and similarities of photocatalysis and electrocatalysis. Then, the traps of catalytic-related systems in 2D nanomaterials will be emphasized to direct related experiment processing to consider and exclude several details for all-round research. Moreover, an introduction of typical 2D nanocatalysts that have long been considered research hotspots will be exhibited, including their classification, structures, synthesis approaches and characterizations. The catalytic applications of 2D nanomaterials in the areas of environmental treatment and biochemical technologies will be discussed. Finally, opportunities, challenges and development directions for 2D catalysts for photocatalysis and electrocatalysis will be described. The intention of this review is to inspire and direct interest in this research realm for the creation of future 2D nanomaterials for photocatalysis and electrocatalysis.

**Table 1:** Comparison of differences and similarities for photocatalysis and electrocatalysis in 2D nanomaterials

		Photocatalysis	Electrocatalysis
Characteristics of catalysts	Type	2D nanomaterial powders or composite thin films	2D nanomaterial thin films or powders loaded on an electrode surface
	Hydrophilicity	Hydrophilic	Hydrophobic or hydrophilic
	Recyclability	Complex for powders, feasible for thin films	Feasible
Conditions of reaction systems	Energy input	Solar energy	Externally applied electric bias
	Configuration	One reaction chamber	One reaction chamber or two chambers separated by membrane
	Charge transfer pathway	Relatively short	Relatively long
Benefits	Cost	Low	High including expensive electrode and electric energy
	Efficiency	Low	Relatively high

## Strategies for Catalytic Performance Enhancement of 2D Nanomaterials

**Fig. 1:**
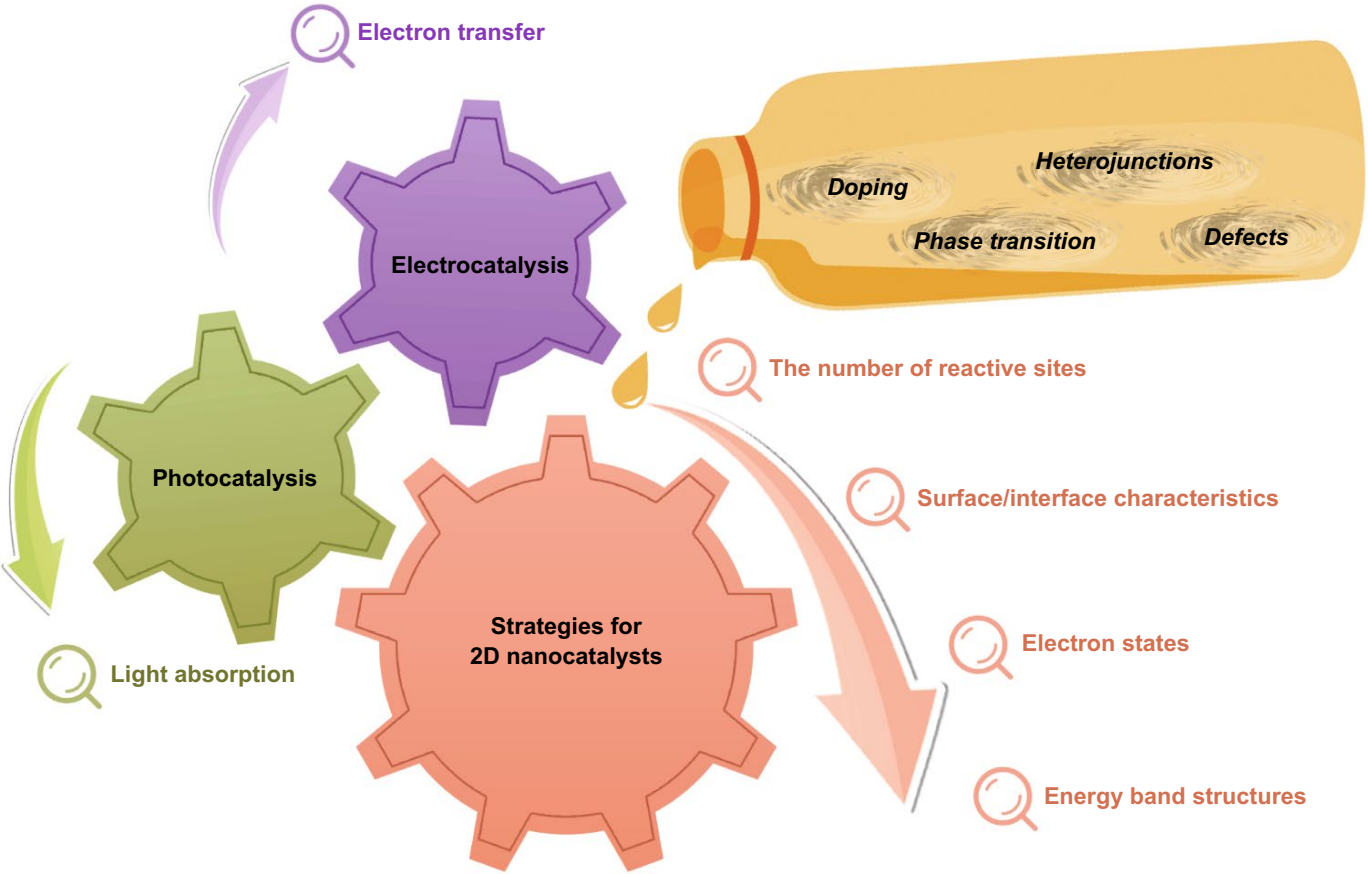
A series of general strategies for 2D nanocatalysts cover the number of reactive sites, surface/interface characteristics, electron states and energy band structures. Specific strategies for photocatalysis and electrocatalysis are, respectively, on the basis of light absorption and electron transfer. Besides, approaches, such as doping, heterojunctions, phase transition and defects, perform the function as lubricants to realize the above-mentioned strategies for catalytic performance enhancement

Carrier separation and transfer kinetics are generally considered as the most significant issues for improving performance for photocatalysis and electrocatalysis [45], which can be considerably related to structure–activity correlation of catalysts [46, 47]. To date, 2D layered nanomaterials including graphene and graphite-like materials continue to suffer from a variety of issues that restrict their functionality and practicability in applications related to semiconductors, sensors and catalysis [37]. Therefore, diverse and abundant strategies must be explored and analyzed to produce layered nanomaterial-based catalysts with enhanced photocatalysis and electrocatalysis performances. Obviously, 2D nanocatalysts show a variety of advantage contrasted to 3D bulk catalysts, which will be presented in the following subsection. In addition, differences and similarities in strategies of photocatalysis and electrocatalysis will be, respectively, discussed and all of these contents are concluded in Fig. 1.

### Superiority of 2D Nanocatalysts

Structure–activity correlation of 2D nanocatalysts illustrates a significant influence of geometric configurations in catalytic performance [46–48], which can be attributed to the unique characteristics 2D nanocatalysts possessing as follows:

1. Surface-active sites. The general geometric configurations of 2D nanocatalysts result in large specific surface area, which can lead to large exposed lattice planes and surface-active sites with high density for further. High density surface-active sites can enhance the catalytic reactions on the material surface. Another approach to increase the exposure of surface-active sites is to decrease 2D nanocatalysts’ lateral size [49]. For example, it is reported that ultrasmall molybdenum disulfide (MoS_2_) exhibits enhanced hydrogen evolution reaction (HER) performance than bulk MoS_2_, which can be attributed to an enrichment of active sulfur edges for HER [50].
2. Carrier mobility. High electron mobility has been extensively observed in various ultrathin 2D nanomaterials, including graphene, transition metal dichalcogenides (TMDs) and black phosphorous (BP) nanosheets [30, 51]. For instance, the reported mobility of graphene and MoS_2_ ranges in 10^2^ ~ 10^4^ cm^2^ V^−1^ s^−1^ [52–56] and around 10^1^ cm^2^ V^−1^ s^−1^ [57–61], respectively. The unique ultrathin structure of 2D nanocatalysts provides relatively high charge migration due to short transport path and small basic resistance. Yu et al. observed an obvious drop of HER performance when MoS_2_ attached by additional atomic layer, which can be interpreted as the restriction of electron mobility along a vertical direction between the material layers [62].
3. Energy band structures. For a variety of 2D layered nanomaterials, changing number of layers in the crystal can tune the band gap, where the tunable band structure determines the light absorption properties of materials for photocatalysis. The band gap of MoS_2_ can be tuned via changing the number of cumulate layers, where the band gap of single-layered and few-layered MoS_2_ are 1.8 ~ 1.9 and 1–1.2 eV, respectively [63]. The reported band gap of g-C_3_N_4_ can be tuned ranging from 1.6 to − 1.1 eV versus normal hydrogen electrode (NHE) [64]. For bismuth-based 2D layered nanomaterials, the band gap can be manipulated ranging in 0.3–3.6 eV via introducing various cations and anions into intrinsic structure, whose corresponding light response covers ultraviolet to near infrared [65, 66]. In addition to the light absorption properties, the interfacial adsorption/desorption free energy between catalysts and reactants can also be regulated via the tunable band structure as well as the changing electron distribution [67].
4. Electronic properties. Through controlling the thickness of 2D nanocatalysts can realize the regulation of the electronic properties [68]. As reported, the electronic structures of 2D nanomaterials are able to regulate the bond strength between reactants and catalytic active sites and reduce the desorption kinetic barrier [69]. Fang’s group reported a battery based on 2D mesoporous covalent organic frameworks (COFs) with superior areal capacitance, gravimetric power and maximum power density of 5.46 mF cm^−2^, 55 Kw kg^−1^ and 4.1–5.4 W cm^−3^, respectively; this was two orders of magnitude better than conventional Li thin-film batteries [70]. Atomic ultrathin 2D nanocatalysts bring the benefits of abundant in-plane defects that are conducive to the electronic properties such as electrical conductivity [71], which improves the conduction of electricity energy sources generated during catalysis. Voiry et al. reported abundant defects of monolayered tungsten disulfide (WS_2_) nanosheets are beneficial for HER, which related to the induced lattice distortions [72].
5. Mechanical properties. 2D nanomaterials prone to possess prominent mechanical properties [73–75], which confer high catalyst durability that is a path to practical application for benefit of humankind. In addition, the robustness of 2D nanomaterials offers the possibility in the development of hybrid nanocatalysts for catalytic enhancement.

On the basis of the above concluded superiority of 2D nanocatalysts, great progress has been achieved in 2D nanomaterials for photocatalysis and electrocatalysis [10, 34, 43, 44]. Nevertheless, the catalytic activity of 2D nanomaterials needs to be further enhanced, and there are a series of strategies gradually emerging [76–78], which will be suggested in the following subsections.

### Similarities in Strategies of Photocatalysis and Electrocatalysis

For various strategies of photocatalysis and electrocatalysis in 2D nanomaterials, there are differences as well as similarities in specific catalytic systems. Herein, we will discuss the similarities in strategies of 2D nanocatalysts’ photocatalysis and electrocatalysis.

In general, the similar strategies of 2D layered nanocatalysts for both photocatalytic and electrocatalytic enhancement can be concluded both kinetically and thermodynamically through regulating the following guidelines, such as (1) the number of reactive sites, (2) surface/interface characteristics, (3) electronic properties and (4) energy band structures, where these common guidelines can be promisingly realized via regulating morphology, doping, constructing heterostructures, importing defects and engineering phases, etc.

1. The number of reactive sites. As above-mentioned in the last subsection, 2D nanocatalysts with a unique morphology of ultrathin atomic layers bring the benefits of the highest number of catalytic reactive sites according to the highest surface area in theory. Set 2D layered MoS_2_ nanosheets as an example, an enhanced electrocatalytic HER ability has been achieved by morphologically controlling the surface structure with the regulation of size and thickness to expose more electrocatalytic reactive sites [35]. For photocatalytic degradation reactions, Parzinger et al. demonstrated that monolayered MoS_2_ nanosheets’ edges sites were more resistant than those of multilayered MoS_2_ nanosheets [79]. In addition, the catalytic reactive sites can be induced via doping, and different doping atoms always generate desperate catalytic sites [10]. In the case of graphene catalysts, the electrocatalytic reactive sites for oxygen reduction reaction (ORR) in B-doped graphene are B atoms [80, 81], but those of N-doped graphene are C atoms next to N dopants [82]. In addition, improved photocatalytic performance has been obtained in B-doped and P-doped g-C_3_N_4_ nanosheets [83–85].
2. Surface/interface characteristics. In general, the basal planes of most 2D nanocatalysts are inertial [76]. A typical example is TMDs. As exhibited by many experiments, doping is able to effectively activate the S sites on the original inertial surfaces for catalysis via introducing Fe, Co and Ni atoms in TMDs [86, 87]. This phenomenon can be attributed to reduced antibonding states [88]. As reported, the Δ*G*_H*_ of Co-doped MoS_2_ nanosheets dropped to 0.1 eV from the original 0.2 eV of MoS_2_ nanosheets [89]. Constructing heterostructures is another significant protocol for controlling surface/interface characteristics to enhance catalytic activities, according to the complex chemical bonds at the interfaces of disparate nanomaterials [77, 90, 91]. At the meanwhile, synergistic interactions regulate the surface/interface properties via physically adjusting confined electron transfer. For instance, Qiao’s group composited g-C_3_N_4_ and N-doped graphene and realized an enhanced electrocatalytic HER, as a result of the heterostructure structures and their synergistic interactions are beneficial to the proton adsorption/reduction kinetics at the surfaces/interfaces [77]. Besides, Tu et al. observed an obvious enhanced photocatalytic simultaneous reduction-hydrolysis in a hybrid structure of TiO_2_-graphene nanosheets, whose Ti^3+^ on the surface can prevent the recombination of electron–hole pairs during the production of methane (CH_4_) and ethane (C_2_H_6_) [92].
3. Electronic states. For all catalytic reaction systems, electronic states determine the separation and transport of electric carriers, which will have a large impact on practical catalytic performance [34]. In general, high level of carrier transport mobility benefits to these electric carriers moving to the catalytic reactive sites. Xie and the co-workers modulated the electronic structures and raised the intrinsic conductivity of MoS_2_ electrocatalysts through the ways of constructing controllable disordered structures and oxygen doping, which demonstrated excellent electrocatalytic HER [93]. In a two-dimensional catalyst, the electronic states of defects and their adjacent regions are often different from those of other parts without defects [94]. Take graphene as an example, density functional theory (DFT) calculations have been performed to support defects enriched at zigzag edges of nanomaterials, which exhibited distinct electronic density of states that were react actively for electrocatalytic ORR [71, 95, 96]. In addition, the electronic states of 2D nanocatalysts can be altered by lattice strain as well, which can optimize catalysis performance for further [97–99]. It was reported that lattice-strained 1 T WS_2_ nanosheets performed enhanced HER, which owned crystal lattices with large deformation and ~ 3% high local strain regions [72].
4. Energy band structures. As generally known, the light absorption properties of 2D nanomaterials for photocatalysis are largely dependent on energy band structures [100], where photoinduced related discussions will be analyzed in the next subsection for the specificity in strategies of 2D nanomaterial-based photocatalysis. In addition to the controlling for light absorption properties, energy band structures typically regulate redox potentials to drive electric carrier dynamics for catalysis reactions including photocatalysis as well as electrocatalysis [101–103]. Additionally, the phase transition of 2D nanocatalysts can also regulate the energy band structures as well as related electronic states for controlling catalytic performance [104]. Take TMDs as example, through the approach of lithium intercalation for monolayered MoS_2_, WS_2_ and tungsten selenide (WSe_2_), their band gap structures were regulated to improve the charge transfer kinetics, and their electronic properties exhibited metallic [105]. As a result, the electrocatalytic HER performance could be enhanced within the phase transition from 2H to 1 T [105]. In addition to the modified band gap structures according to the improved layer spacing, the oxidation states of Mo and W decreased with the regulation of d-band filling, on the basis of the experimental results [104].

Generally, the above-mentioned four aspects are the most common strategies for catalytic performance enhancement covering photocatalysis and electrocatalysis, where they are correlative instead of independent of each other; as a result, they are always conditioned together.

### Differences in Strategies of Photocatalysis and Electrocatalysis

The biggest differences between photocatalysis and electrocatalysis are diverse driving ways for redox reactions that photoinduced electric carriers and external circuit-induced carriers dominate the catalytic reaction processing, respectively.

**Fig. 2:**
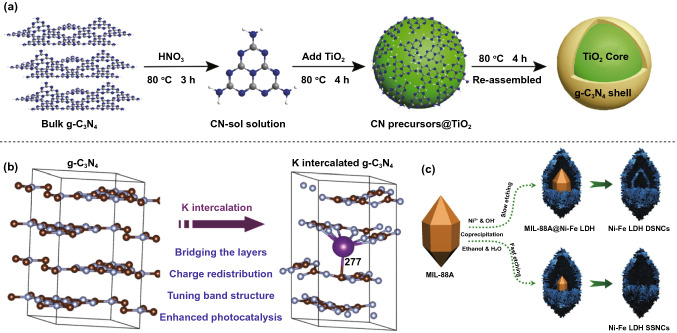
Strategies for catalytic activity enhancement. **a** Core–shell structures of g-C_3_N_4_@TiO_2_ promote carrier separation. Reproduced with permission [121]. Copyright 2018, Elsevier. **b** K-doped g-C_3_N_4_ nanomaterials to achieve enhanced visible-light absorption, efficient carrier separation and strong oxidation capability. Reproduced with permission [136]. Copyright 2016, American Chemical Society. **c** Ni–Fe LDH nanocages with regulated tunable shells perform optimal chemical composition possessing large electroactive surface area. Reproduced with permission [155]. Copyright 2020, Wiley–VCH

For photocatalysis, the development of light absorption catalysts is a key goal for photocatalysis [6], but conventional photocatalysts suffer from an uncontrolled extinction coefficient and severe photocorrosion during irradiation by sunlight; these factors result in poor catalytic performance and limited practical applications [106]. For example, as a representative all-organic semiconductor material, a layered g-C_3_N_4_ has been widely reported in catalytic-related applications [107–111]. However, pure g-C_3_N_4_ nanomaterials continue to suffer from secondary pollution, limited visible light absorbance and high electron–hole recombination, which remain significant challenges for the development of highly efficient catalysis [112–116]. To meet these challenges, a variety of g-C_3_N_4_-based composite catalysts with core–shell structures have been reported in an effort to improve the photoresponse to visible light and carrier separation [117–120]. The core–shell structures of hybrid catalysts promote the separation of photoinduced carriers in disparate components, resulting in an enhanced photocatalytic performance. Wang et al. reported on a sol–gel synthesized few-layered g-C_3_N_4_@TiO_2_ core–shell nanocomposite catalyst for efficient visible light photocatalysis, where the layers could be finely controlled through the regulation of the colloidal suspension concentration and calcination temperature, as shown in Fig. 2a [121]. In this case, TiO_2_ makes up for the lack of light absorption and photoresponse of the layered g-C_3_N_4_ nanomaterials, while the generated chemical bonds of the g-C_3_N_4_ shell and TiO_2_ core benefit for photoinduced carrier separation [121]. In addition, the doping of layered g-C_3_N_4_ nanomaterials, as an effective modification strategy, is able to regulate the electronic structure to control the light responsive range and improve carrier separation [110, 120, 122–128], where the S, B, F and C atoms replace lattice atoms, and transitional metals are incorporated into the framework [129–135]. Xiong et al. designed a unique electronic structured K-doped g-C_3_N_4_ nanomaterial and achieved an excellent enhancement in photocatalytic nitric oxide (NO) removal performance, as shown in Fig. 2b [136]. According to the DFT calculations, the use of K intercalated doping with a specific structure of K atoms that can bridge the layers leads to a narrowing of the bandgap of g-C_3_N_4_, thereby leading to decreased electronic localization, positively shifted valence band position and an enlarged π conjugated system. As a result, the K-doped g-C_3_N_4_ nanomaterial provides an enhanced visible light absorbance, effective carrier separation and strong oxidizing property [136].

Take COFs as example, most photocatalysis-related research based on COFs is in the field of the reduction in carbon dioxide and production of hydrogen; however, there are few reports of the use of COFs for photocatalytic treatment of toxic organic pollutants in wastewater. In addition, the disadvantages of high cost, harsh synthesis conditions and long reaction time make COFs less economically viable for practical applications such as environmental wastewater treatment. This is probably a result of the COFs structure being formed by covalent bonds, in contrast to metal organic frameworks (MOFs) which are formed by coordination bonds. In addition, the most significant challenges for the creation of new COFs catalysts are their low chemical stability, low catalytic efficiency and low cost-efficiency, where a series of strategies for synthesis and design approaches are needed; this includes a need for excellent chemical stability, strong catalytic activity and high cost-efficiency. As a result, COFs are often combined with other materials, such as TiO_2_, MoS_2_, cadmium sulfide (CdS), zinc sulfide (ZnS), cadmium selenide (CdSe) and graphene, to enhance visible light absorption, promote electric carrier transfer efficiency and increase specific surface area [137–140]. This is achieved by taking advantage of its large conjugated structure that is conducive to electron transport and strong visible light absorption. It is worth highlighting that another strategy for optimizing COFs is to establish favorable electron donor–acceptor characteristics, for example by using active metal nanoparticles to provide improved charge separation and a broadening of the absorption range [141–146]. As a result, a range of optimized COFs matrix composites with excellent photocatalytic activity has been reported to provide efficient treatment of organic pollutants in wastewater.

For electrocatalysis, electrochemical water splitting has promising capacity for hydrogen and oxygen production; however, the oxygen evolution reaction is limited due to a non-negligible overpotential and depressed reaction kinetics [147–149]. In general, active sites for oxygen generation are located on the catalyst surface; thus, a large surface area of the catalyst is desirable for excellent catalytic performance [13, 78, 148]. Ni–Fe-layered double hydroxides (LDHs) have been reported as an excellent oxygen evolution catalyst in an alkaline solution due to synergistic interactions between Fe and Ni [150–154]. The subtle design of nanomaterial structure can be considered to be one of the most significant strategies for catalysis reaction enhancement for electrocatalytic applications [155]. Zhang et al. synthesized Ni–Fe LDH nanocages with regulated tunable shells, and realized noble electrocatalysis for the oxygen evolution reaction, where Ni–Fe LDH materials with an optimal chemical composition possessed a large electroactive surface area, as shown in Fig. 2c [155]. In addition, the process of sonication can modify the LDH hydrophobic surface in a xylene solution and break the LDH into small fragments with an average lateral size that is decreased to dozens of nanometers [156].

To sum up, there are abundant strategies that have been explored and analyzed for photocatalytic and electrocatalytic activity enhancement in 2D nanomaterials, which can be generally concluded as the number of reactive sites, surface/interface characteristics, electron states and energy band structures; however, specific strategies for photocatalysis and electrocatalysis are, respectively, on the basis of light absorption and electron transfer. The above-mentioned strategies can be achieved via the approaches, such as doping, heterojunctions, phase transition and defects.

## Traps of Catalytic Systems in 2D Nanomaterials

**Fig. 3:**
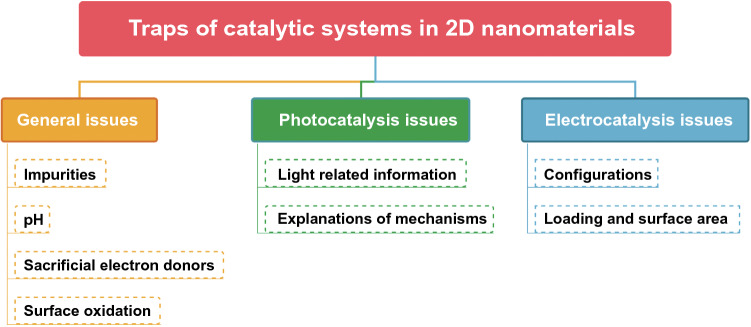
A brief summary for general and special traps of different catalysis systems in 2D nanomaterials. General issues include impurities, pH, sacrificial electron donors and surface oxidation. Specific photocatalysis issues include light-related information and explanations of mechanisms. Specific electrocatalysis issues include configurations and loading and surface area

As researchers gradually in-depth research in the research field of photocatalysis and electrocatalysis, there are increasing publications focused on catalytic performance enhancement of 2D nanomaterials. Most of the catalytic-related laboratory works, ultrahigh catalytic behaviors are desirable, but these probably seem to be a series of traps under the laboratory circumstances, due to rare 2D nanocatalyst products possessing long-term stability in efficient catalytic activity in reality. Besides, each research group draws up specific rules for their own catalytic reaction systems including the experimental parameters of additive amount, external energy input and environmental implication. The specificity of catalytic system designing is hard to avoid the yielding of a series of traps during the experimental processes according to such freedom. These traps can be considered as details that are easy to be overlooked during the catalytic reaction systems, which may lead to improved catalytic activities. In this section, we will give a brief summary for general and special traps of different catalysis systems in 2D nanomaterials, which also can be seen in the dendrogram of Fig. 3.

For general catalysis, experimental environment and 2D nanocatalyst-related issues are the most factors that are able to be possible traps in experimental catalysis systems. First, the impurities from the catalytic reaction systems are able to largely influence the final catalytic performance including the purity of 2D nanocatalysts and the contamination of glassware, especially when the reaction product yield at a relatively low level. For instance, the generation and deposition of metallic impurities from the counter electrode during the catalytic reaction may give the catalytic possibility for materials originally incapable of catalytic activity [157, 158]. When the catalytic solutions are extremely alkaline, the generation of silicates from glassware could have an impact on the results of the catalytic experiments [159]. Second, in addition to the resulting generation of impurities from surroundings, the solution pH is considered as a key role in controlling protonated states of reactants; as a result, the pH parameters should be disclosed when writing articles. For example, the formation of bicarbonate when catalytic carbon dioxide (CO_2_) reduction reactions can decrease the pH of the solutions [160]. Third, during catalytic CO_2_ reduction activities and hydrogen evolution, sacrificial electron donors are always added for the promotion of reductive reactions [161], but the product yields may impact when introducing sacrificial electron donors. For example, when catalytic CO_2_ reduction reactions processing, organic sacrificial electron donors, such as ethylene diamine tetraacetic acid (EDTA) and triethanolamine (TEOA), may be oxidized, which will further generate C_1_ [162]. Similarly, the oxidation of alcohols and SH^–^ can evolute H_2_ during catalytic hydrogen evolution reactions [163]. Fourth, surface oxidation of 2D nanocatalysts for photocatalysis and electrocatalysis is common phenomenon, where the formation of hydroxide or metal oxide surface layer could protect the catalyst for stable catalytic activities [164]. Herein, it is of great significance to make the real substance clear through surface-sensitive techniques before and after the catalytic reactions.

For photocatalysis, the photocatalytic performance-related performances, covering the parameters of quantum yields, energy efficiencies and reaction rates, depend largely on photon flux, wavelength and scattering [165]. Therefore, these light-related information as well as nanocatalyst’s light absorption are key factors for photocatalysis that must be considered and mentioned in the manuscript, but till now less photocatalytic-related papers involve the information. This undoubtedly makes it difficult for other researchers to repeat and improve the experiments. In addition, the explanations of mechanism for photocatalytic systems are limited in hydroxyl radical-mediated reactions and interfacial electron transfer processes. However, simple assumption according to previous work is far from enough to prove the photocatalytic mechanisms, due to every photocatalytic reaction system is unique [166]. As a consequence, more analysis is needed to rule out the irrelevant explanations for specific photocatalytic reaction systems.

When conducting electrocatalytic experiments, specific electrochemical configurations are of great importance, such as three-electrode or two-electrode configurations. For three-electrode configurations, there will be large deviation for electrocatalytic products of counter electrode compartment, if the counter electrode’s potential does not keep a set level. For electrocatalytic reaction systems possessing the separating membrane between different compartments, the drop of voltage for membrane structure should be considered rigorously [167]. Similarly, when reporting overpotential, it is important to carefully consider relevant configuration issues [168]. In addition to configuration issues, the loading and surface area of 2D nanocatalysts also exhibit impact on the specific electrocatalytic overpotential; as a result, the information of 2D nanocatalyst loading and morphological-based surface area must be provided in the manuscript, but a great deal of literatures are not available [169].

In this section, we have discussed the general and special traps of different catalysis systems in 2D nanomaterials. General issues include impurities, pH, sacrificial electron donors and surface oxidation. Specific photocatalysis issues include light-related information and explanations of mechanisms. Specific electrocatalysis issues include configurations and loading and surface area.

## D Nanocatalysts

In Sect. 4, we will briefly introduce typical 2D nanocatalysts that have long been considered research hotspots for general catalytic applications, through discussing their classification, structures, synthesis approaches and characterizations in turn as the following subsections.

### Classification of 2D Nanocatalysts

The development of atomically thin 2D graphene nanomaterials has propelled progress in related ultrathin 2D nanomaterials [12]. In general, most 2D nanomaterials can be sketchily classified into layered materials [44], where van der Waals interactions between layers make layer stacking, and continuous atom layers within layers are typically strong chemical bonded [170]. Typical 2D layered nanomaterials for catalysis include graphene, graphitic carbon nitride, a family of mono-elemental compounds, TMDs, COFs, metal carbides and nitrides (MXenes), LDHs, bismuth-based layered compounds, hexagonal boron nitride (h-BN) MOFs and 2D metal nanomaterials, etc.

The structure of graphite was determined with the advent of single-crystal X-ray crystallography, where *graphene* is generally considered as an atomically thin single-layer graphite crystal [171]. Atomically thin graphene nanomaterials are promising materials with a performance that can exceed conventional semiconductors for catalytic applications [12, 37]. However, graphene nanomaterials are a form of zero band gap semi-metal [172]; therefore, they are usually considered as a co-catalyst or an effective catalyst support, rather than a catalyst directly [173].

One of the most well-known structural graphene-like 2D nanomaterials is g-C_3_N_4_ [107]. Generally, g-C_3_N_4_ nanomaterials are able to act as a potential catalyst in numerous redox reactions due to their chemical inertness under strong acid or alkali environments [64]. However, challenges such as high electric carrier recombination rate, low specific area and poor mass transfer can restrict the efficiency of catalysis, where approaches such as heterojunction coupling, surface defect engineering and element doping have been considered to address this deficiency [124].

*Pnictogens* are mono-elementals of group VA that exhibit a high energy and power density and can be produced by stacking layered materials of various characteristics to create a heterostructure to combine the superior aspects of each material [174]. As a typical family of mono-elemental compounds, an ultrathin 2D structure of BP in the orthorhombic phase was first synthesized in 1914 [175]. Compared to graphite, black phosphorus can enhance the specific capacity from 372 to 4200 mAh g^−1^, with a reversible reaction with Li and Na [176–179]. Moreover, their thermodynamically stable properties for electronic applications enable operation at extreme temperatures and at humidity in air, which results in efficient and stable catalytic reactions [180–184].

TMDs generally consist of chalcogen atom layers, with a transition metal atom interlayer [185, 186]. Changing the number of layers in the crystal provides an opportunity to regulate the band gap of TMDs. MoS_2_-based nanomaterials, as a typical TMD material, exhibit unique lattice vibration properties, high catalytic activity, low cost and natural abundance [63]. To date, 2D layered MoS_2_ nanomaterials have demonstrated significant potential to replace graphene nanomaterials in a variety of applications due to their unique characteristics. Zhang et al. utilized MoS_2_ to achieve outstanding catalytic properties for the N_2_ reduction, compared to other catalysts reported under the same circumstances, where the Faradaic efficiency and the NH_3_ yield rate reached high levels of 1.17% and 8.08 × 10^–11^ mol s^−1^ cm^−2^, respectively [187].

COFs are formed from organic ligands through reversible covalent bonds and are considered as advanced crystalline porous materials. In 2005, Yaghi and co-workers provided the first demonstration of connecting small symmetric organic structural units to a covalent organic skeleton of a porous crystal using the principle of dynamic covalent chemistry [188]. Tan’s group explored a green and facile approach for creating a 2D heterogeneous P6-Au-COF hybrid nanomaterial that was formed using a COF and pillar[6]arene reduced Au nanoparticles (P6-Au), which showed high catalytic performance for the reduction in nitrophenol isomers [189].

Graphene-like MXenes have been synthesized from stacks of scrolls and sheets, including mono-transition-metal MXenes and double-transition-metal MXenes [190–198]. Monolayered MXenes show metallic properties due to their high electron state concentration near the Fermi level [192, 199–202]. A high electron state concentration near the Fermi level indicates that MXenes are potential layered materials for catalytic applications [191]. MXenes possess excellent electronic conductivity, high elastic moduli and good hydrophilic properties that have been exploited in a variety of applications such as hybrid electrochemical supercapacitors and Li-ion battery anodes [194, 200, 203–212].

LDHs are constructed using brucite-like host layers and interlayered structural water with positive charges and negatively charged anions, respectively [213, 214]. LDHs, especially those that contain transition metals, are widely reported to be a promising catalyst with a high catalytic activity for applications related to the generation of oxygen and hydrogen [215]. Zhang’s group reported on ultrathin NiFe-LDH nanosheets with a 0.6 nm thickness and achieved an overpotential of 254 mV for the electrocatalytic water splitting reaction, and demonstrated superior charge transfer properties [216].

Bismuth, as an environmentally friendly metal, possess a wide range of interesting features for a range of applications, including catalysis [217–221]. Bismuth-based 2D layered nanomaterials have been reported to demonstrate high performance for energy conversion and storage devices [222–227]. The band gap can be manipulated from 0.3–3.6 eV, by introducing a variety of cations and anions into the intrinsic structure, whose corresponding light response spans the ultraviolet to near infrared [65, 66]. Besides, the effective mass and mobility of photoexcited electric carriers are restricted and improved, which is beneficial to applications such as optoelectronic energy conversion, photodetection and photochemical catalysis [228, 229].

The h-BN is a hexagonal crystal system with a graphite-like hierarchical structure. It has been utilized as a catalyst carrier or catalyst as a result of its high temperature resistance, high thermal conductivity of ~ 390 W m^−1^ K^−1^, extremely stable chemical properties, strong acid corrosion resistance, and high electrical insulation. Nevertheless, due to its low electrical conductivity, attempts have been made to functionalize the h-BN monolayer by combination with electrically conductive materials such as reduced graphene oxide (rGO) [230, 231] and carbon nanotubes (CNTs) [232], which can expand its range of applications [233].

Yaghi et al. reported a coordination compound which was synthesized from rigid organic ligands and a transition metal with a 2D structure, as a MOF [234]. MOFs exhibit a single-layered lamellar structure which is one-atom thick, resulting in high aspect ratio and the possibility of post-synthetic modification that can help realize tailor-made pores for selective adsorption and catalysis and the incorporation functional groups into the MOFs [235, 236]. Since transition metals represent a large proportion of the MOF, it is beneficial to provide large pore dimensions, large surface area and a versatility of the type of MOF structure formed.

2D metal nanomaterials, especially noble metals [237], are synthesized as the forms of nanosheets [238–240], nanodisks [241], nanoplates [242, 243], nanoribbons [244], nanorings [245] and nanobelts [246], etc. Due to the interesting electronic and structural properties, 2D metal nanomaterials have been applied in a variety of catalysis [71, 247, 248]. Huang et al. reported 2D Pd nanosheets performed large enhancement in electrocatalytic formic acid oxidation reaction (FOR) in comparison with commercial Pd black [238], attributed to abundant active sites on the catalyst surface [247].

### Structures of 2D Nanocatalysts

**Fig. 4:**
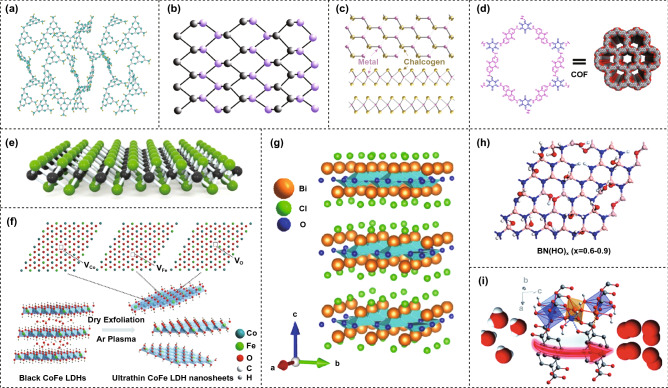
Example 2D nanomaterial structures. **a** Graphene and graphitic carbon nitride. Reproduced with permission [260]. Copyright 2015, Wiley–VCH. **b** Family of mono-elemental compounds. Reproduced with permission [263]. Copyright 2018, Springer Nature. **c** TMD. Reproduced with permission [264]. Copyright 2017, American Chemical Society. **d** COF. Reproduced with permission [189]. Copyright 2020, IOP Publishing Ltd. **e** MXene. Reproduced with permission [191]. Copyright 2017, Wiley–VCH. **f** LDHs. Reproduced with permission [271]. Copyright 2017, Wiley–VCH. **g** BiOX. Reproduced with permission [275]. Copyright 2020, American Chemical Society. **h** h-BN. Reproduced with permission [276]. Copyright 2014, American Chemical Society. **i** MOF. Reproduced with permission [277]. Copyright 2020, Royal Society of Chemistry

In the last subsection, we have discussed the classification of 2D nanomaterials. These 2D nanomaterials possess distinct crystal phases related to atomic coordination, atomic arrangement and layered stacking [97, 249, 250], which can largely regulate the properties and catalytic activities [105, 251–253]. Here, we will discuss the structures of the above-mentioned 2D nanocatalysts, where a range of 2D catalytic nanomaterials with distinct structures is highlighted in Fig. 4.

Graphene comprises hexagonal or honeycomb-like geometry carbon atoms in *sp*^2^-hybridized form, where every carbon atom connects with the adjacent three atoms via *σ*-bond covalently bonding [254].

The planar structure of g-C_3_N_4_ is specifically different from that of graphene, where carbon and nitrogen atoms constitute N-substituted graphite frameworks in *sp*^2^-hybridized form [255–257]. In general, g-C_3_N_4_ possesses two typical structures that are formed by tri-*s*-triazine units and *s*-triazine units [255, 258, 259]. For g-C_3_N_4_ with tri-*s*-triazine units, at a temperature of 900 K in vacuum, the structure of the g-C_3_N_4_ monolayer becomes disordered, where the hydrogen bonds within NH/NH_2_ groups are broken, resulting in NH/NH_2_ groups twisting outward, as seen in Fig. 4a [260]. Such amorphous g-C_3_N_4_ nanomaterials can achieve enhanced photocatalysis for hydrogen generation in contrast to the crystalline form of g-C_3_N_4_ when illuminated by visible light [260].

The layered puckered honeycomb structural BP exhibits an orthorhombic crystal and a space group of *Cmca*. Each P atom connects with the adjacent three P atoms, in which three locate at the same plane, but the rest one atom locates at another plane [261, 262]. Figure 4b illustrates the typical structure of BP from a top view [263].

Monolayered TMDs stack together to form layered TMDs via van der Waals interactions in general. Individual monolayered TMD is made of one sandwiched transition metal atomic layer and two chalcogen atomic layers [24]. The structure of a TMD is shown in Fig. 4c which indicates both the top and side views, where the chalcogen atoms and the metal atoms are bonded covalently with trigonal prismatic coordination [264].

Periodic porous COFs are orderly formed by organic building block units with covalent connection [265, 266]. A typical structure of a COF is illustrated in Fig. 4d [189], which has the advantages of a high level of inherent porosity, adjustable aperture, good conjugation structure, large surface area, crystallizability, no secondary pollution and wide visible light response range.

Layered MXenes (M_n+1_X_n_T_x_) can be gained via selective etching treatment for A-group elements from parent layered ternary carbides (M_n+1_AX_n_, MAX) [194–198], where MAX possesses hexagonal structure with a space group of *P6*_3_/*mmc* [267]. Geng et al. achieved notable catalytic hydrogen evolution reactions using a Mo_2_C-on-graphene MXene heterostructure, where the crystal structure of Mo_2_C can be seen in Fig. 4e [191].

The general formula of LDHs is [M_1-x_^2+^M_x_^3+^(OH)_2_] (A^n−^)_z/n_·yH_2_O, where the marks of M^2+^, M^3+^ and A^n−^ represent divalent metal cation, trivalent metal cation and interlayer anion, respectively. A^n−^ locates in the hydrated interlayer gap, while M^2+^ and M^3+^ locate octahedral holes in the brucite-like layer [268–270]. Wang et al. synthesized ultrathin CoFe LDH nanosheets for use as a highly efficient oxygen evolution electrocatalyst, where the Ar plasma exfoliation fabrication process and the variety of structures formed can be seen in Fig. 4f [271].

Bismuth-based 2D layered nanomaterials achieve high-dispersion band due to *s-p* hybridization and anisotropic *p*, and thus, the photogenerated carriers exhibit low effective mass and electron–hole pairs with high mobility [272–274]. In particular, bismuth oxychloride (BiOCl) is a typical bismuth-based layered nanomaterial with a tetragonal structure and a P4/nmm space group. The electronic properties and lattice dynamics of this material have been reported, and the layered crystal structure is illustrated in Fig. 4g, where the Bi and O atom layers are sandwiched between the Cl atom layers [275].

H-BN is a hexagonal crystal system with a graphite-like hierarchical structure, whose crystal structure can be seen in Fig. 4h [276]. Normally, h-BN nanosheets are formed from *sp*^2^ hybridized B atoms, along with N atoms that are regularly arranged in a hexagonal ring network between the individual layers [276]. In a similar manner to most layered nanomaterials, the B and N atoms are tightly covalently bonded within the layer planes. Moreover, the weak van der Waals interactions of the interlayer bonding are beneficial for material exfoliation to create ultrathin nanosheets [12].

In general, MOFs can be considered as the metal–organic skeleton materials that are self-assembled via coordination bonds between metal ions/clusters and organic ligands. The organic ligands in MOFs are called the *linkers*, and the metal ions or clusters are called the *nodes*, which are then self-assembled into coordination compounds with periodic structures. Li and the co-workers constructed 2D layered MOFs from LDHs via a facile ligand-assisted procedure, where the material exhibited a superior performance for water oxidation, which are illustrated in Fig. 4i [277]. However, the electrical conductivity of 2D MOFs is relatively poor, below 10^−14^ S cm^−1^ [278, 279], as a result of the internal porosity due the stacking of several atomic layers [280]. In addition, 2D MOFs suffer from a high sensitivity to humidity and structural instability; this is due to the weak coordination bonds that are located between the metal nodes and linkers [280, 281].

### Synthesis of 2D Nanocatalysts

**Fig. 5:**
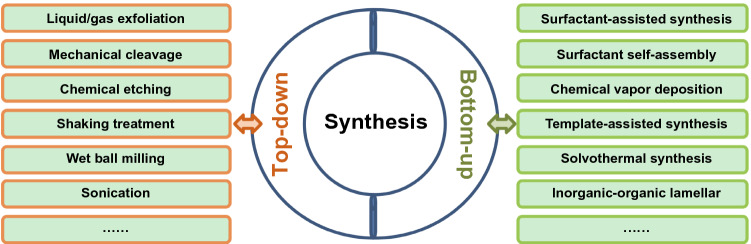
Common synthesis procedures for 2D nanomaterial based on top-down and bottom-up methods. Top-down methods include liquid/gas exfoliation, mechanical cleavage, chemical etching, shaking treatment, wet ball milling, and sonication. Bottom-up approaches include surfactant-assisted synthesis, surfactant self-assembly, chemical vapor deposition, template-assisted synthesis, solvothermal synthesis and inorganic–organic lamellar

The *top-down* and *bottom-up* methods are regarded as the two primary procedures for the synthesis of 2D layered nanomaterials synthesis, and the general approaches are summarized in Fig. 5. These approaches include a variety of common methods which are based on top-down and bottom-up methods.

Top-down synthesis is generally considered as a demixing processes of layered bulk materials by the external forces [282]. The key to the approach is the breaking of weak interlayered van der Waals interactions and achieve the cleavage of bonds along the layer plane to obtain 2D ultrathin nanosheets [280]. There are a number of approaches for synthesizing 2D nanomaterials via top-down methodologies, these include liquid/gas exfoliation [283], mechanical cleavage [284, 285], shaking treatment [286], wet ball milling [287, 288], sonication [216, 289] and chemical etching [287]. In the following discussion, we will discuss common approaches for top-down 2D layered nanomaterial synthesis.

*Liquid exfoliation* methods have been considered as one of the most popular ways for the preparation of multiple 2D layered nanomaterials, whose synthesis mechanism is based on weakening interlayer interactions by introducing guest molecules to enlarge the spacing of interlayers, and interfacial debonding for the formation of a steady sol via ultrasonic processing [290]. Zhang’s group utilized N-methyl-2-pyrrolidone solutions for exfoliation of few-layer black phosphorus nanosheets to synthesize 2D layered electrocatalysts and achieved high electrocatalytic performance for oxygen generation applications [24, 226].

The *mechanical cleavage* method is also a common procedure for the preparation of multiple 2D layered nanomaterials. Subbiah and Jayasena utilized an ultrasharp diamond wedge that was assisted by ultrasonic oscillations to exfoliate 2D graphene layers with an approximate area of 300 × 900 μm^2^ [285]. The well-known Scotch tape method, regarded as one of most common mechanical exfoliation methods [24], led to the discovery of graphene [291] and is able to prepare high-quality 2D monolayered nanomaterials with desirable performance. For example, Fuhrer’s group demonstrated a large single crystal of diindium triselenide (In_2_Se_3_) exhibits strong optical properties, which was formed by exfoliation in a similar way to graphite using sticky tape (3 M Scotch) with a thickness of ~ 100 nm [284].

The *wet ball milling* approach is a method of grinding materials, solvents and zirconia spheres into nanometer slurry at a certain ratio. Yang’s group reported on the synthesis of ultrathin layered MOFs with high crystallinity, large lateral area and 1 nm thickness, via a wet ball milling and ultrasonic exfoliation procedure, where the rotation speed was at a low level of 60 rpm, and propanol and methanol were used for ultrasonication exfoliation [288].

Although top-down methods benefit the preparation of 2D nanomaterials with exceptional properties using a straightforward approach to provide a low cost product [286], these methods continue to exhibit limitations which limit the use of 2D nanomaterials for practical applications. These include the formation of unstable nanosheets, uncontrollable layer numbers, poor homogeneity, being limited to layered materials, low product yield and the stripped nanosheets often break up and restack. Hence, we believe basic research of top-down 2D nanomaterial synthesis in needed, where progress on strategies and development will expectedly improve more effort [292], such as intercalation-assisted expansion and exfoliation [293–296], and exfoliation of layer materials containing ions or molecules between the layers [192, 293].

The bottom-up synthesis for 2D layered nanomaterials can be generally considered on the basis of the anisotropic assembly of small molecules with growth limitations in vertical directions [297]. The bottom-up wet chemical synthesis approaches are easier to realize for large-scale production and provides a more controllable synthesis of 2D nanomaterials. These include surfactant-assisted synthesis [298], surfactant self-assembly [299], chemical vapor deposition [300–303], template-assisted synthesis [304], inorganic–organic lamellar [305] and solvothermal synthesis [306, 307]. The following discussion will discuss typical methods for 2D nanomaterials synthesized using bottom-up methods.

Lang and co-workers synthesized atomic layered binary MOF nanosheets via a bottom-up solvothermal method and achieved excellent electrocatalysis for oxygen generation with a solvent based on a N,N-dimethylacetamide solution [307]. Chemical vapor deposition is a popular approach for large-scale 2D material production with promising superiority in a controllable size and thickness of material for practical applications [308]. Ji’s group present a chemical vapor deposition-based approach for the synthesis of 2D black phosphorus, with average areas generally over 3 μm^2^. Song’s work demonstrated chemical vapor deposition growth of high-quality h-BN nanomaterials with a thickness that typically ranged from two to five atomic layers. In this work, ammonia borane was used as the precursor for the BN, followed by a gas flow of Ar/H_2_ at a temperature of around 1000 ℃s, with a typical growth time of 30–60 min [300].

However, traditional bottom-up synthesis strategies often require the aid of substrate materials and surfactants. It is therefore difficult to prepare dispersed 2D nanomaterials, and the residual surfactants in the products are difficult to remove, which can limit their applications.

### Characterization of 2D Nanocatalysts

In the above subsections, we have discussed the classification, structure, synthesis of 2D nanocatalysts. In addition to these topics, the advances in characterization technologies propel the rapid development of 2D nanomaterials for related application in catalysis as well [44, 309]. So far, there have been a series of sophisticated characterization technologies for 2D nanomaterials, including multiple optical, electron and probe microscopies and various spectroscopies, that can uncover material information of morphologies, defects, crystal phase, electron density of states and so on [310–313]. In this subsection, we will briefly introduce several typical characterization technologies for distinguishing 2D nanomaterials.

The optical microscopy (OM) can rapidly provide the location and morphology information of materials [314]. By the utilization of OM, Chiu et al. demonstrated that the stacking layers of MoS_2_/WSe_2_ heterostructures exhibited a clear distinction with color contrast [315]. However, more accurate value of material morphology information needs much preciser characterization such as electron and probe microscopies. Scanning electron microscopy (SEM) is the most common technique for structure, topology and morphology characterization of nanocatalysts, whose resolution generally achieves several nanometers [316]. Transmission electron microscopy (TEM) is another strong technique for morphology, crystallinity and phase characterization of nanocatalysts; besides, it can always be combined with selected area electron diffraction (SAED) patterns to get more crystallinity information [317]. Jung et al. fabricated a CO_2_ reduction composite photocatalyst TiO_2_-graphene-MoS_2_ and observed the composite structure and morphology through SEM, the crystallinity and phase information through TEM, respectively [318]. More accurate thickness information and electronic properties of 2D nanocatalysts can be obtained by scanning probe microscopy (SPM) [319], such as atomic force microscopy (AFM) [320], scanning tunneling microscopy (STM) [76], kelvin probe force microscopy (KPFM) [321] and electrostatic force microscopy (EFM) [322]. Heath’s group utilized monolayered graphene to visualize water adlayer on mica with the average height of 0.37 ± 0.02 nm [323]. Jaramillo et al. controlled different active sites of MoS_2_ electrocatalysts, and identified these active sites via STM; as a result, the HER electrocatalytic activity showed a linear relation with the amount of catalytic active sites [76]. Additionally, EFM was used to analyze the electrostatic screening effects of MoS_2_ atomically thin layers [322].

Apart from advanced microscopies, various sophisticated nondestructive spectroscopies are also used for characterization of 2D nanomaterials, including Raman spectroscopy [324], X-ray photoelectron spectroscopy (XPS) [72] and X-ray absorption spectroscopy (XAS) [325]. Raman spectroscopy provides spatial resolution and high spectral in the electronic and structural information of 2D nanomaterials [326]. XPS is able to distinguish different crystal phases within 2D nanocatalysts [327]. Voiry et al. calculated quantitatively each phase concentration of WS_2_ nanosheets with the help of XPS [72]. XAS can characterize the atomic-scale structural information, including species of the atoms, coordination chemistry and oxidation states [328]. Sun et al. proposed a pits-confined CeO_2_ nanosheet platform for catalytic CO oxidation evaluation at a variety of active catalytic centers, where it indicated by XAS that the average coordination number of pit-surrounding cerium sites was 4.6 when the artificial CeO_2_ nanosheets with ~ 20% pits occupancy [325].

In Sect. 4, we have overviewed the classification, structure, synthesis and characterization of 2D nanocatalysts. The classification and structure have been discussed in a range of 2D nanocatalysts including graphene, graphitic carbon nitride, a family of mono-elemental compounds, TMDs, COFs, MXenes, LDHs, bismuth-based layered compounds, h-BN, MOFs and 2D metal nanomaterials. The common synthesis procedures for 2D nanomaterial are based on top-down and bottom-up methods. Top-down methods include liquid/gas exfoliation, mechanical cleavage, chemical etching, shaking treatment, wet ball milling, and sonication. Bottom-up approaches include surfactant-assisted synthesis, surfactant self-assembly, chemical vapor deposition, template-assisted synthesis, solvothermal synthesis and inorganic–organic lamellar. In addition, we have introduced a series of typical characterization technologies for 2D nanomaterials covering the microscopies of OM, SEM, TEM, SPM and the spectroscopies of Raman, XPS, XAS.

## Catalytic Applications of 2D Nanomaterials

In this section, we provide a discussion on the photocatalytic and electrocatalytic applications of 2D nanomaterials based on recent publications, which are mainly focused on environmental treatment and biochemical technologies including dye degradation, elimination of toxicant, HER, oxygen evolution reaction (OER), carbon dioxide reduction reaction (CO_2_RR) and cancer therapy. In addition, Table 2 summarizes the variety of 2D nanomaterial-based electrocatalysts applied for specific electrocatalytic reactions for practical applications, along with synthesis methods, applied conditions, detailed electrocatalytic performance and basic catalysis mechanisms.

**Table 2:** Overview of range of 2D nanomaterials for electrocatalytic applications

2D nanomaterials	Synthesis	Application	Conditions	Performance or parameter	Activity origin or mechanism	Refs
Double-gyroid MoS_2_	Electrodeposition, followed by sulfidization	HER	Acidic medium	Overpotential = 150–200 mV Tafel slope = 50 mV decade^−1^	Surface embellishment for edge site exposure	[35]
Co_3_S_4_	Physical etching	HER	Alkaline medium	Ƞ_10_ = 63 mV Tafel slope = 58 mV decade^−1^	Abundant sulfur vacancies	[340]
Co–N-GA	Solvothermal	HER	Acidic medium	Onset = 0 V, Ƞ_10_ = 46 mV Tafel slope = 33 mV decade^−1^	Synergetic effect of N-doped carbon and inner metal Co	[306]
MoS_2_	Hydrothermal	HER	Acidic medium	Ƞ_-200_ = 198 mV Tafel slope = 36 mV decade^−1^	Facilitated ion diffusion by channel engineering	[36]
WSe_2_	Hydrothermal	HER	Acidic medium	Onset = 150 mV Tafel slope = 78 mV decade^−1^	Many exposed edge sites can provide abundant active reaction sites	[186]
Mo_2_CT_x_	Ball milling, HF etching	HER	Acidic medium	Ƞ_10_ = 189 mV Tafel slope = 70 mV decade^−1^	T_x_ as surface functional groups	[287]
NiFe-LDH	Ultrasonication	OER	Alkaline medium	Ƞ_10_ = 254 mV Tafel slope: 32 mV decade^−1^	Metal and oxygen vacancies	[216]
Ni–Fe-MOF	Solvothermal	OER	Alkaline medium	Ƞ_10_ = 221 mV Tafel slope = 56 mV decade^−1^	Fe constitutes the active site	[307]
CoFe LDH	Hydrothermal and Water-plasma-enabled exfoliation	OER	Alkaline medium	Ƞ_10_ = 232 mV Tafel slope = 36 mV decade^−1^	As-exfoliated increased active sites and multi-vacancies	[215]
CoCo-LDH	Soft template method	OER	Alkaline medium	Ƞ_10_ = 319 mV Tafel slope = 42 mV decade^−1^	More highly active edge sites with lower coordination number and mass diffusion promotion	[346]
Ni(OH)_2_	Chemical etching	OER	Alkaline medium	Ƞ_10_ = 335 mV Tafel slope = 65 mV decade^−1^	Holes developed inside the sheet structure supply tremendous permeable channels for ions adsorption and transportation	[344]
BP	Liquid phase exfoliation	OER	Alkaline medium	Onset = 1.45 V, Ƞ_10_ = 300 mV Tafel slope = 88 mV decade^−1^	Reduction in thickness generates active sites and improves specific surface area	[283]
Ni-MOF@Fe-MOF	Ultrasonication	OER	Alkaline medium	Ƞ_10_ = 265 mV Tafel slope = 82 mV decade^−1^	Hybridization and cooperativity between Ni and Fe	[289]
Co-C_3_N_4_/CNT	Polycondensation reactions, and acid leaching process	ORR and OER	Alkaline medium	HER onset = 0.9 V, OER onset = 1.5 V Tafel slope = 68.4 mV decade^−1^	M-N_2_ coordination	[171]
Fe-Co/N-rGO-Al	Solvothermal	ORR	Alkaline medium	Onset = 0.98 V half-wave potential = 0.84 V	Four electron transfer mechanism and a lower HO^2−^ yield	[347]
Pd	Thermal treatment	ORR	Alkaline medium	Mass activity (0.85 V) = 21.1 mA mg^−1^ electron transfer number = 3.73–3.85	Unique structural features	[237]
WSe_2_	Chemical vapor transportation	CO_2_RR	Acidic media	Current density = 18.95 mA cm^−2^ CO formation turnover frequency = 0.28 s^−1^ Overpotential = 54 mV	Presence of ionic liquids and high density of edges	[349]
Ru/MgAl	Wet impregnation	CO_2_RR	Gas phase reaction	CO_2_ conversion = 85% CH_4_ yield = 84%	Non-thermal plasma (NTP) activated CO_2_ hydrogenation	[348]

Today, we all use a wide variety of medicines and personal care products, leading to irreversible damage as they enter the ecosystem [329]. Antibiotics and their metabolites have potential toxicological risks with regard to non-resistant microorganisms, phytoplankton, fish and other aquatic organisms that may disrupt the aquatic food chain [330]. They are difficult to fully degrade by traditional biological processes, and their toxicity may be further amplified as they accumulate in our aquatic ecosystems [331]. Therefore, there is a need to explore advanced technologies that are able to destroy organic compounds in aquatic environments. Here, we will discuss the recently reported catalytic related applications in environmental treatment and biochemical technologies using 2D nanomaterials.

### Dye Degradation

**Fig. 6:**
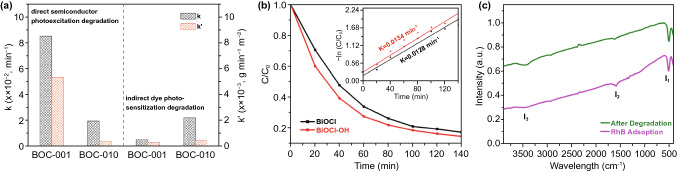
Catalytic degradation applications of 2D nanomaterials. **a** Direct semiconductor photoexcitation pollutant degradation in single-crystalline BiOCl nanosheets under UV light. Reproduced with permission [332]. Copyright 2012, American Chemical Society. **b, c** BiOCl and BiOCl-OH photocatalyst for UV light driven photocatalytic dye degradation. Reproduced with permission [333]. Copyright 2017, American Chemical Society

**Fig. 7:**
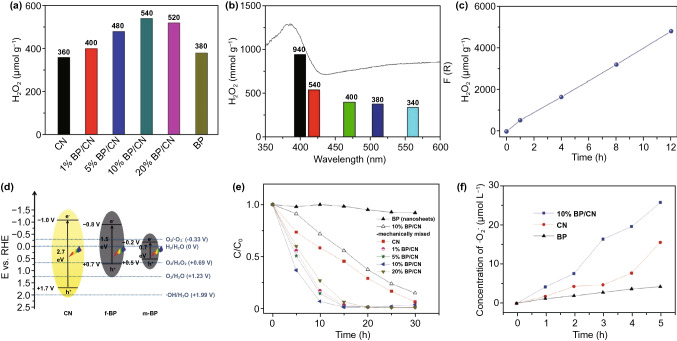
Layered heterostructure structured black phosphorous/graphitic carbon nitride (BP/CN) nanomaterials for efficient photocatalysis. Reproduced with permission [334]. Copyright 2018, Wiley–VCH

With regard to dye degradation, it should be emphasized that 2D nanomaterials are suited for oxidative dye photodegradation reactions since their small kinetic barriers and optimal thickness provide a high surface area [39]. For example, Zhang and co-workers synthesized highly crystalline BiOCl single-crystalline nanosheets through a hydrothermal method [332]. Figure 6a demonstrates photoexcitation degradation in BiOCl nanomaterials, and the direct semiconductor degradation efficiencies were 99% and 59% in BiOCl-001 and BiOCl-010, respectively. An indirect semiconductor exhibited a lower photocatalytic activity compared to direct semiconductor, and the BiOCl nanomaterials demonstrated higher photoexcitation performance under UV light than under visible light. In addition, Yang et al. constructed BiOCl and BiOCl-OH photocatalysts for efficient photocatalysis and degradation of Rhodamine B dye in wastewater illuminated by UV light at a wavelength of 365 nm, as illustrated in Fig. 6b, c [333]. The BiOCl-OH exhibited an enhanced photocatalysis compared to pure BiOCl since the UV light induced increased the number of oxygen vacancies, and the peaks in the FT-IR spectra correspond to hydroxyl groups and indicate the significant role of hydroxyl groups in the photocatalytic activity for Rhodamine B dye degradation [333]. Zheng et al. developed a layered hetero-structured black phosphorous/graphitic carbon nitride (BP/CN) nanomaterial to obtain efficient photocatalysis for dye degradation and produce the highly reactive oxygen species of H_2_O_2_, as shown in Fig. 7 [334].

### Elimination of Toxicants

Phenolic compounds are highly toxic organic pollutants that pollute water. These compounds are derived from pharmaceutical, printing, dyeing, pesticide and oil refining industries. The presence of phenols in industrial sewage makes surface water extremely vulnerable to pollution. The search for efficient and safe degradation technologies is therefore worthy of worldwide attention [37]. For example, Liu and co-workers found that a hetero-junctioned photocatalysts based on g-C_3_N_4_/Bi_2_WO_6_/rGO (incorporated with 3 wt% of rGO) exhibited a 86% and 98% reduction in ibuprofen by photocatalytic degradation under optical light and solar light irradiation, respectively [335].

Antibiotics are able to cause irreversible damage due to their easy accumulation in the human body, and photocatalytic oxidation has been applied as an efficient approach to antibiotics removal for wastewater treatment. Norvill and co-workers demonstrated that the antibiotic tetracycline could be reduced by 93% and 99% with a biomass concentration and chemical oxygen demand (COD) at hydraulic retention times of 4 and 7 days under summer-like conditions, although the lower photodegradation during the winter can lead to a reduced overall removal efficiency [336]. These results are the first to provide an effective demonstration of tetracycline removal in an outdoor wastewater environment and demonstrate that algal wastewater treatment provides a higher removal capacity compared to conventional biological wastewater treatment.

### Hydrogen Evolution Reaction (HER)

**Fig. 8:**
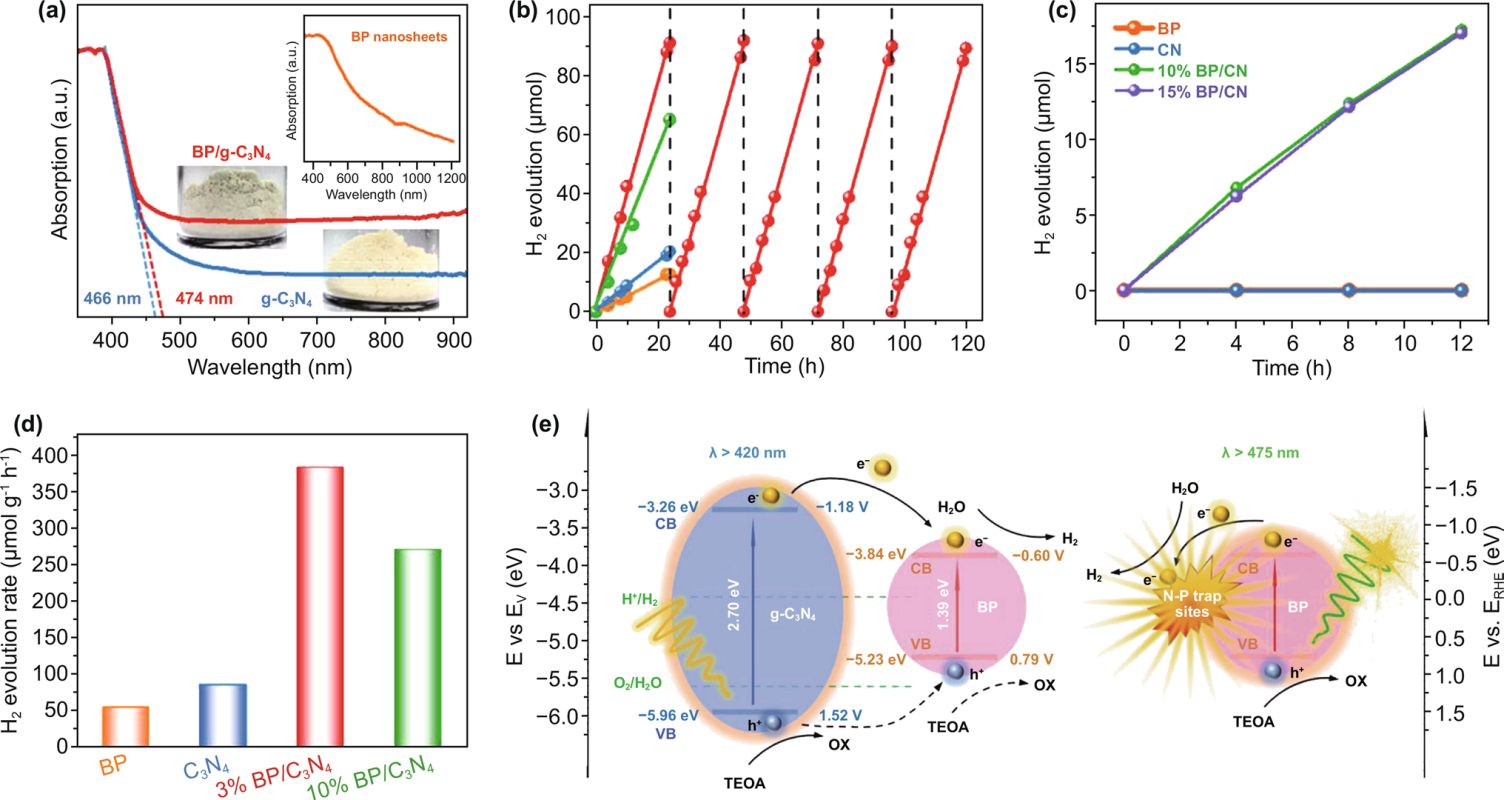
BP/g-C_3_N_4_ nanosheets with properties of high-quality, large lateral size and lower anomalous structural defects for high efficiency catalytic H_2_ production. Reproduced with permission [183]. Copyright 2019, Wiley–VCH

Today, there is a need to develop new energy under the current worldwide circumstances of increasing environmental pollution and the energy crisis [337–339]. Hydrogen energy, as one of new and clean energy resources, possesses not only no secondary pollution, but also has a high energy density and has emerged as a low-carbon and zero-carbon energy. The HER is the cathodic reaction that can be described as 2H^+^  + 2e^−^ → H_2_, known as the half part of water splitting [340]. For more detailed processes of the HER in acidic solutions, it can be divided into two main procedures including proton adsorption and hydrogen desorption, whose basic mechanisms are on basis of the Volmer mechanism, the Heyrovsky mechanism and the Tafel mechanism, where the adsorption sites of electrocatalysts play a significant role for the HER [12]. Numerous research on developing 2D material catalysts is exploring their attractive physicochemical properties as a potential catalyst for efficient HER activity with high efficiency [341]. For example, Ma and co-workers prepared ice-assisted exfoliated BP/g-C_3_N_4_ nanosheets from bulk black phosphorous, which exhibited the properties of high product quality, a low density of anomalous structural defects and large lateral size [183]. Figure 8 illustrates the corresponding properties of the synthesized BP/g-C_3_N_4_ nanosheets. The absorption spectra for individual BP, g-C_3_N_4_ and BP/g-C_3_N_4_ nanosheets indicate that the absorption band of the BP nanosheets ranges broadly in the UV, visible and NIR regions, while the absorption edges of g-C_3_N_4_ and BP/g-C_3_N_4_ nanosheets are ~ 466 and ~ 474 nm, respectively. In addition, the differing component ratios for BP/g-C_3_N_4_ nanosheets have been analyzed, where the BP/g-C_3_N_4_ nanosheets provide a clear photocatalytic enhancement for the HER compared to individual BP and g-C_3_N_4_ catalysts, in terms of both photocatalytic hydrogen generation rate and total amount of product. The existence of BP enlarges the absorption band of the BP/g-C_3_N_4_ and the addition of g-C_3_N_4_ not only preserves the BP against oxidization, but it also provides a shallow interface of trapped charge sites for promotion of electric carrier separation in the composite photocatalysts, which reduces the limitations of fast carrier recombination in the BP or g-C_3_N_4_ nanosheets.

### Oxygen Evolution Reaction (OER)

**Fig. 9:**
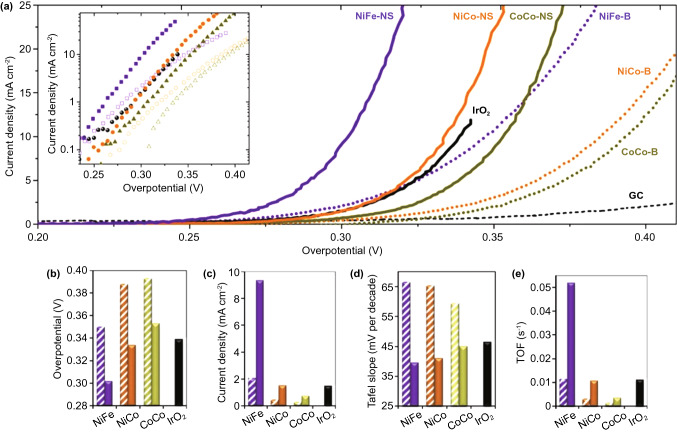
Layered double hydroxide (LDH) nanosheets for catalytic oxygen evolution reaction (OER) enhancement. Reproduced with permission [345]. Copyright 2014, Springer Nature

**Fig. 10:**
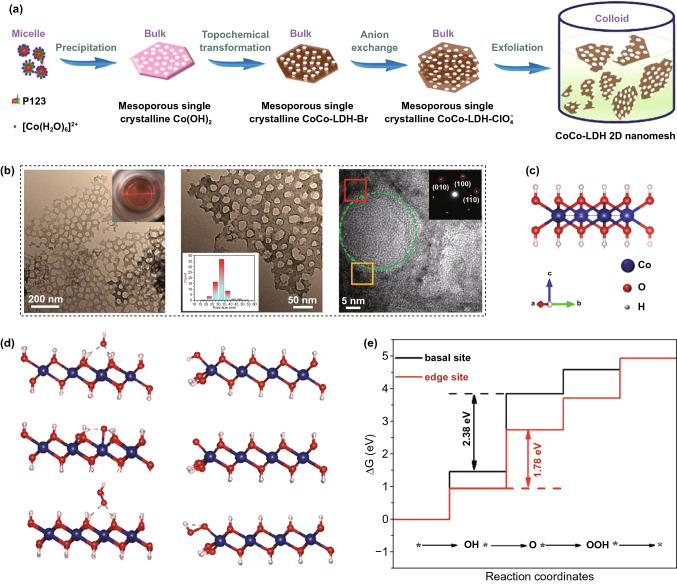
CoCo-LDH 2D nanomesh for enhanced oxygen evolution. Reproduced with permission [346]. Copyright 2019, Wiley–VCH

As the other half part of water splitting, the OER can be regarded as an oxidative reaction that demands four electrons and proton transfer, resulting in an overpotential requirement and a kinetically sluggish response [342–344]. On account of its atomic level thickness, large specific area and large amount of surface atoms, 2D LDH nanosheets are able to realize a significant improvement in catalytic performance [343]. Song et al. presented an orthogonal approach for catalytic OER enhancement using layered LDH nanosheets which were processed via liquid phase exfoliation [345]. As can be seen in Fig. 9, the bulk-layered LDHs exhibit a lower OER performance compared to exfoliated single-layer LDH nanosheets. In addition, Qin et al. synthesized a 2D CoCo-LDH nanomesh as an OER electrocatalyst, where there were abundant high activity atoms with low ligancy, and the mesoporous structure of the CoCo-LDH nanomesh improved the diffusion of reactants and products, as illustrated in Fig. 10 [346]. The onset overpotential and the overpotential (*η*_10_) of the CoCo-LDH nanomesh were decreased to 220 mV and 319 mV, respectively.

### Carbon Dioxide Reduction Reaction (CO_2_RR)

**Fig. 11:**
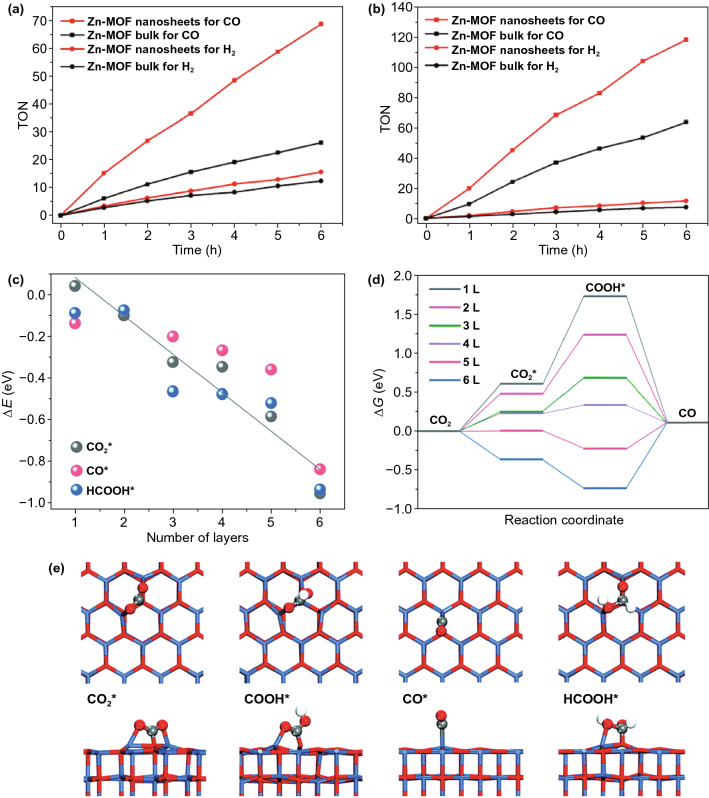
2D nanomaterials for efficient photocatalytic CO_2_ conversion systems. **a, b** Ultrathin 2D Zn-MOF nanosheets for photoreduction of CO_2_ to CO. Reproduced with permission [350]. Copyright 2018, Elsevier. **c-e** 2D ZnO nanomaterials for selective photoreduction of CO_2_. Reproduced with permission [351]. Copyright 2019, Royal Society of Chemistry

Currently, scientists have found that the CO_2_ concentration in the atmosphere surpasses the previous level of 23 million years and is increasing at an unprecedented rate [347]. CO_2_ is considered to be one of the most potent greenhouse pollutants, and the increase in CO_2_ concentration is closely related to climate change [348]. The capture and efficient use of CO_2_ is an urgent global problem [349]. As a result, 2D layered nanomaterials have attracted effort in terms of photocatalysis and electrocatalysis for CO_2_RR applications to transform CO_2_ into nontoxic organics [350]. Ye and co-workers realized an efficient CO_2_RR with a CO_2_ adsorption capacity of 103.8 cm^3^ g^−1^ for homogeneous Zn-MOF nanomaterials with a 4.7 nm layer thickness, as shown in Fig. 11a, b [350]. In comparison with bulk MOFs of low efficiency, the synergistic effect of prolonged lifetime of photogenerated electric carriers offers the possibility of using 2D layered MOF nanosheets with desirable catalytic CO_2_RR activity. Zhao’s group exploited 2D ZnO for photocatalytic CO_2_RR, as shown in Fig. 11c-e [351]. Compared with their bulk counterpart, the 2D ZnO nanosheets have the advantages of desirable bandgap, optical absorbance and large surface catalytic active sites for CO_2_RR.

### Cancer Therapy

**Fig. 12:**
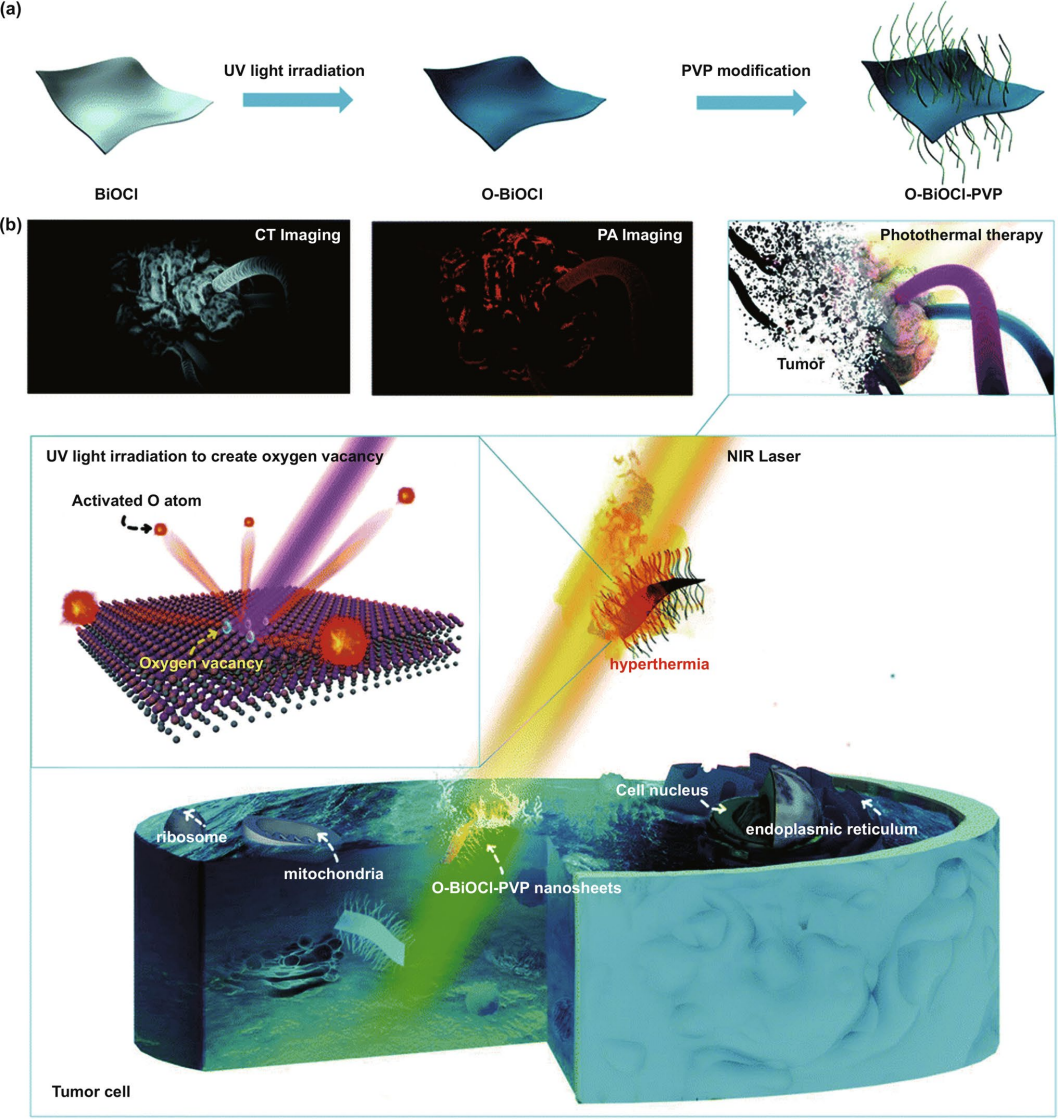
2D BiOCl nanosheets for photonic tumor ablation with the combination of physiochemical properties and photocatalytic effect. Reproduced with permission [364]. Copyright 2020, Royal Society of Chemistry

During recent decades, cancer is one of great threats to humankind and societal health, which inspires the development of functional nanomaterials with desirable characteristics and suitability for cancer therapy [352–361]. Du’s group demonstrated cancer therapy by using 2D nanomaterials based on rare-earth metals [362, 363]. Dai et al. realized noble ablation of tumors that was attributed to the combination of physiochemical properties and photocatalytic effect in hydrothermally synthesized 2D O-BiOCl-PVP nanosheets, where the oxygen vacancies were constructed on material surfaces via UV light irradiation, as illustrated in Fig. 12 [364]. This recent work provides a new direction for defect engineering strategy of nanomaterials and enlarges the biomedical applications of 2D layered nanomaterials.

Here, various catalytic applications of 2D nanomaterials have been discussed in this section that contain environmental treatment and biochemical technologies including dye degradation, elimination of toxicant, HER, OER, CO_2_RR, as well as cancer therapy.

## Future Perspective and Challenges

We now provide an overall perspective on recent progress in 2D nanomaterials for photocatalytic and electrocatalytic applications. This begins with the range of structures and synthesis approaches. We then focus on the emerging strategies for improving catalytic properties by enhancing light absorption ability, increasing reactive sites, accelerating carrier separation and charge migration, and improving surface reaction. Their applications in the realm of environmental treatment and biochemical technologies are highlighted.

Compared with traditional bulk nanoscale catalysts, 2D layered nanomaterials in the fields of photocatalysis and electrocatalysis exhibit specific advantages such as an exposure to more active sites, being more conducive to reactant diffusion and a larger specific surface area. In addition, much effort in optimizing these materials have been devoted to enhancement of stability, electrical and mechanical properties through changing layer thickness, surface modification and external stimulation. Research progress and future directions in 2D nanocatalysts are aiming to tackle a range of issues and challenges, which are summarized below:

1. The macroscopic and controlled production of 2D nanomaterials is key to the practical application of catalysis. Chemical vapor deposition and liquid phase dissection are potential synthesis approaches for the production of 2D layered nanomaterials. Recently, in situ characterization equipment has been developed that is able to detect thermodynamic and kinetic reactions during material synthesis, which can be highly beneficial for developing a detailed understanding of the growth mechanisms of 2D nanomaterials. In addition to existing progress in the manufacture of 2D nanocatalysts within a controlled microenvironment, the use of confined synthesis could be a significant growth approach that is able to operate at a molecule level to offer precise control for the synthesis for 2D nanomaterials for catalytic applications. In the longer term, the large-scale preparation of controllable structures of non-layered atomic thickness nanosheets with intrinsic catalytic activity remains to be developed, which faces significant challenges, especially in achieving precise control for the production of high-quality and homogenous 2D nanomaterials.
2. In general, catalytic activity is closely related to the structure and surface characteristics of a 2D nanocatalyst, which can adjust the electronic properties and electron transfer. The structure and surface characteristics of 2D nanomaterials is related to its dimensions (interlayer spacing, thickness and transverse dimensions), exposed surface atom density, the existence of surface impurities, additional functional groups and surface energy states. It is worth highlighting that the abundant edges of 2D nanomaterials lead to a high electron transfer activity, compared to basal planes. Thus, the design of 2D catalytic nanomaterials can benefit from the regulation of defects, heteroatom-doping and the tuning of edges and planes.
3. The creation of 2D nanomaterials with hybrid composite structures has become topic of intense research interest, where two or more compounds with a different degree of anisotropy and characteristics create new possibilities in the design of 2D nanocomposites with multi-functional and tailored properties for catalysis applications. In addition, heterostructures that are built by combining individual materials are showing promising potential in providing control of structure and electronic properties. Future directions for catalytic-related applications in 2D nanomaterials could therefore involve the creation of sandwich structures, confined space structures, and strong electron interactions. In addition, the concept of coupling multiple systems provides new opportunities to enable multiple mechanisms to operative cooperatively. For example, piezoelectric semiconductors possess piezoelectricity and photovoltaic effects simultaneously. By combining these two effects, the photocatalytic activity can be enhanced by an internal piezoelectric field that couples both piezoelectric and photovoltaic effects.
4. With regard to developing a greater theoretical understanding of the mechanisms of catalysis in 2D nanomaterials, there have been anastomotic models that link experimental results and theoretical analysis. However, current catalytic studies of 2D nanomaterials have placed an emphasis on catalytic activity enhancement, rather than the underlying science of the catalytic mechanisms, and much of research on 2D nanocatalyst-based catalysis involves trial and error. In addition, the use of 2D layered nanocatalysts makes the reaction system more complex compared to 3D bulk materials; thus, new developments in the underlying science of 2D catalytic nanomaterials are beneficial for material design and discovery. For example, with regard to photocatalysis, the bandgap of a semiconductor for catalysts using light excitation is a dominant factor. The fundamental mechanisms and specific impact of the dimensionality of 2D nanomaterials and 3D bulk materials to control the energy bandgap positions remain unclear. To date, the theoretical catalytic mechanisms of 2D nanomaterials are still not easily applicable to real complex reaction systems, thus more theoretical and fundamental studies on catalytic mechanisms are worthy of study.
5. In addition to technology development for mass production, the ability for catalyst shaping is of interest for end-use applications. When 2D nanomaterials are used in the form of loose powders they can agglomerate which can restrict their application. The potential of fixing 2D nanomaterials to a substrate provides a promising approach for improving their ease of use. For example, 2D nanomaterials could be epitaxially grown on the surface of other materials, be assembled into a foam, or be supported on carbon fiber paper or nickel foam for catalytic activity enhancement.

2D nanomaterials have become one of the most promising forms of catalysts applied to both photocatalysis and electrocatalysis, but it is merely on the threshold for comprehensive analysis both in terms of experimental data and theoretical understanding. By furthering our understanding in 2D nanocatalysts in terms of production, material design, hybridization, catalytic mechanisms, and applications, the potential of 2D nanomaterials for practical catalysis in the future will be more clearly understood. The intention of the review is therefore to inspire new efforts to accelerate the development of 2D layered materials for catalysis-related applications.
